# Plasticity of Intermediate Filament Subunits

**DOI:** 10.1371/journal.pone.0012115

**Published:** 2010-08-12

**Authors:** Robert Kirmse, Zhao Qin, Carl M. Weinert, Andrea Hoenger, Markus J. Buehler, Laurent Kreplak

**Affiliations:** 1 Department of Molecular, Cellular, and Developmental Biology, University of Colorado, Boulder, Colorado, United States of America; 2 Department of Civil and Environmental Engineering, Massachusetts Institute of Technology, Cambridge, Massachusetts, United States of America; 3 Department of Physics and Atmospheric Science, Dalhousie University, Halifax, Nova Scotia, Canada; Indiana University, United States of America

## Abstract

Intermediate filaments (IFs) assembled *in vitro* from recombinantly expressed proteins have a diameter of 8–12 nm and can reach several micrometers in length. IFs assemble from a soluble pool of subunits, tetramers in the case of vimentin. Upon salt addition, the subunits form first unit length filaments (ULFs) within seconds and then assembly proceeds further by end-to-end fusion of ULFs and short filaments. So far, IF subunits have mainly been observed by electron microscopy of glycerol sprayed and rotary metal shadowed specimens. Due to the shear forces during spraying the IF subunits appear generally as straight thin rods. In this study, we used atomic force microscopy (AFM), cryo-electron microscopy (cryo-EM) combined with molecular modeling to investigate the conformation of the subunits of vimentin, desmin and keratin K5/K14 IFs in various conditions. Due to their anisotropic shape the subunits are difficult to image at high resolution by cryo-EM. In order to enhance contrast we used a cryo-negative staining approach. The subunits were clearly identified as thin, slightly curved rods. However the staining agent also forced the subunits to aggregate into two-dimensional networks of dot-like structures. To test this conformational change further, we imaged dried unfixed subunits on mica by AFM revealing a mixture of extended and dot-like conformations. The use of divalent ions such as calcium and magnesium, as well as glutaraldehyde exposure favored compact conformations over elongated ones. These experimental results as well as coarse-grained molecular dynamics simulations of a vimentin tetramer highlight the plasticity of IF subunits.

## Introduction

The term intermediate filaments (IFs) refers to a heterogeneous family of cytoskeletal proteins with the ability to self-assemble into 8–12 nm wide filaments [1]. The self-assembly pathway of IFs, as we currently understand it *in vitro*, is a hierarchical process where subunits stable at high pH and low ionic strength start to merge laterally when the ionic strength is increased and the pH is lowered to neutral [2]. The subunits first form unit length filaments (ULFs) 60 nm long and 15 nm in diameter as seen by electron microscopy (EM) of glutaraldehyde fixed and stained samples [3]. Within seconds most of the subunits are merged into ULFs [4]. Then the assembly proceeds by the end-to-end annealing of ULFs and filaments [5]. The later has also been observed within cells expressing fluorescently tagged IF proteins [6]. As the assembly proceeds, subunits can be observed along side the filaments [7].

Among all IF proteins, vimentin subunits are the most widely studied. Early on, Soellner and coworkers demonstrated that cells of mesenchymal origin contain a soluble pool of a tetrameric subunit in equilibrium with the vimentin IF network [8]. Since then, we have acquired a good understanding of the tetramer architecture through X-ray crystallography of vimentin fragments, EM of glycerol sprayed and rotary metal shadowed tetramers, and analytical ultracentrifugation [5], [9], [10]. The vimentin tetramer appears as a ∼75 nm long rod with moderate flexibility (Figure 2A in [5]). It is in fact an antiparallel, half-staggered arrangement of two 50 nm long double-stranded α-helical coiled-coil dimers [11]. Recent research has resulted in a first structural model of vimentin subunits with atomistic detail, which allows the first structural and mechanical property calculation in combination with experimental deformation tests [12], [13].

Even so cryo-electron (cryo-EM) microscopy and atomic force microscopy (AFM) have been used extensively to image and manipulate ULFs and single IFs in various conditions [7], [14], [15], no experimental data exists on the structural dynamics of any IF subunits. In this study, we used a combination of cryo-EM, AFM and molecular dynamics (MD) simulation [12] to gain further insights on the dynamic properties of vimentin, desmin and keratin K5/K14 subunits. Subunits embedded in vitreous ice appeared as thin straight rods but the use of a molybdate staining solution and/or glutaraldehyde favored more compact conformations and aggregation into meshworks. AFM imaging in air of filaments adsorbed on mica after one hour of assembly were surrounded by subunits displaying a wide range of conformations from dots to flexible rods. The assembly in the presence of divalent ions such as calcium, or glutaraldehyde fixation biased the distribution of conformations towards the most compact ones. MD simulations of a full atomistic single vimentin dimer and tetramer [12] in solution and adsorbed to a coarse-grained mica surface enabled us to map in detail the type of conformations accessible to an IF subunit. Overall, these data indicate that IF subunits are indeed very “plastic” structures that can achieve dramatic shape changes depending on their environment.

## Materials and Methods

### IF proteins preparation

Human vimentin and desmin were expressed and purified as described [16]. The proteins were stored at −80°C in 8 M urea, 5 mM Tris-HCl (pH 7.5), 1 mM DTT, 1 mM EDTA, 0.1 mM EGTA, 10 mM methyl ammonium chloride. The day before use, vimentin was dialyzed into 5 mM Tris-HCl (pH 8.4), 1 mM DTT, at room temperature by lowering the urea concentration in a step-wise fashion (6M, 4M, 2M, 0M). Dialysis was continued overnight at 4°C into fresh buffer without urea. In the desmin case, we used 2 mM phosphate buffer (pH 7.5), 1 mM DTT, instead of the above mentioned Tris buffer.

Human K5/K14 heterodimers were expressed and purified as described [17]. The protein was stored at −80°C in 6 M urea, 10 mM Tris-HCl (pH 8.5), 2 mM DTT, 1 mM EDTA, 0.1 mM EGTA, 10 mM methyl ammonium chloride. The day before use, the proteins were dialyzed into 2 mM Tris-HCl (pH 9), 1 mM DTT, at room temperature by stepwise lowering the urea concentration as described above. Dialysis was continued overnight at 4°C into fresh buffer without urea.

### Assembly conditions

Vimentin, or K5/K14 samples, in their respective dialysis buffer, at a concentration of 0.2 mg/ml were assembled at 37°C for up to one hour after addition of an equal volume of 40 mM Tris-HCl (pH 7) containing different concentrations of either NaCl and CaCl_2_. Desmin samples at a concentration of 0.2 to 0.5 mg/ml were assembled at 37°C for up to one hour after addition of an equal volume of 2 mM phosphate buffer (pH 7,5), 200 mM KCl.

### Cryo-electron microscopy

For cryo negative staining of the IF tetramers we followed earlier published protocols [18], [19] using holey carbon films on copper grids. The staining solution was prepared freshly on the day of use. Briefly it consists of saturated ammonium molybdate/water solution (1.2 g in 0.875 ml) neutralized with 10 M NaOH (0.125 ml to pH 7.2). Right before use the solution, containing slurry of molybdate salt is stirred and after the slurry is sedimented again, the supernatant is used for staining.

Staining of the IF tetramers was performed following the method of De Carlo et al. [20]. The dialyzed IFs where diluted to 0.2 to 0.4 mg/ml and 5 µl were deposited onto the holey carbon grid. After that the grids were placed upside down onto 50 µl aliquots of the molybdate staining solution and incubated for 30 sec. immediately after that the grids were mounted into the plunge freezer, blotted and plunge frozen into liquid ethane. According to [20] the sample in that state is no longer fully hydrated. About 30% water remains per volume. Alternatively samples were prepared for standard cryo-electron microscopy by removing the staining step.

Images were collected on a FEI F20-FEG transmission electron microscope at 200kV acceleration voltage (FEI, Eindhoven, the Netherlands) at a calibrated magnification of 50.000×, utilizing a Gatan 626 cryo-specimen holder (Gatan Inc., Warrendale, PA, USA). The samples were kept at low temperature conditions during data acquisition (ca. −180°C). Electron doses between 1000 and 4000 e^−^/nm^2^ were used.

### Atomic force microscopy

The sample was either diluted or quenched by addition of an equal volume of 0.2% glutaraldehyde in the corresponding assembly buffer. We applied 20 to 30 µl of the obtained solution to a freshly cleaved mica surface for up to 5 min. We flushed the surface with 10 ml of ultrapure water and dried it with Argon.

AFM imaging was performed in acoustic mode at a scanning speed of 1Hz with an Agilent 5500 (Agilent, Santa Barbara, CA) using high frequency (300 kHz) silicon cantilevers with a tip radius of 2–5 nm (TESP-SS, Veeco, Santa Barbara, CA). The amplitude of the cantilever’s oscillations in contact with the surface was set to half of the free amplitude. Images were analyzed using the software Gwyddion (http://gwyddion.net/).

### Coarse-grained molecular dynamics simulations

**Figure 1:**
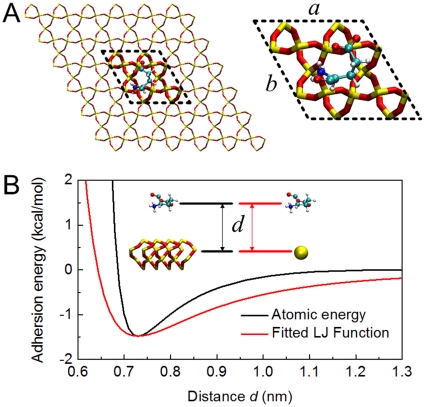
The simulation strategy. A) the full atomic model used to fit the parameter of the coarse-grained substrate model. B) the Lennard-Jones potential for the full atomic model and the coarse-grained substrate model as a function of the distance between two particles.

The simulation system included a full atomistic model of the vimentin subunits (dimer and tetramer) and a coarse-grained model of the substrate (Figure 1). The total energy *E* of the system as a function of the coordinates 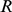 (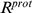 for the coordinates of atoms in protein and 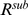 for the coordinates of coarse-grained beads in the substrate) consisted of:
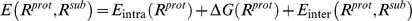
(1)where 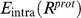 represents the energy of the protein described by the CHARMM19 all-atom energy force field, 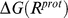 is the solvent free energy given by the effective Gaussian model for the water solvent, and 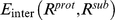 is the non-bonded adhesion energy between the protein and substrate. Both the van der Waals contribution and electrostatic contribution between substrate and protein were considered in this term.

### Coarse-grain substrate

To develop the coarse-grained substrate we took the physical parameters from a silica film and assumed it to be rigid regardless of the applied force (Figure 1A). Each silica crystal cell was represented by a coarse-grained bead. The beads were positioned at the crossing points of a hexagonal lattice with a uniform length of 0.98 nm. The interacting energy was given by:
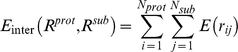
(2)and each pair potential was given by Lennard-Jones potential function as
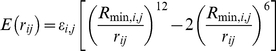
(3)where 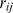 is the distance between the atom pair, 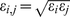 is the energy well depth, 
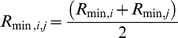
 is the distance of zero force, and 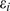, 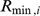 are Lennard-Jones parameters for a specific atom type in the CHARMM force field.

We used the ClayFF-CVFF force field to fit the parameter in this function. Figure 1A shows the intermolecular energy between a unit crystal in the silica layer and a glutamic acid. By considering the depth and location of the lowest well in the measured energy curve we obtained the total adhesion energy well for this amino acid to be 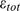 = 1.48 kcal/mol and the zero force distance to be 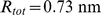 (Figure 1B). We derived 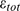 by taking the adhesion energy density 50∼60 mJ/m^2^ [21] and the adhesion area of 0.28 nm^2^ which matches the amino-acid area shown in Figure 1A. This energy was smaller than the one observed for a gecko’s hair adhering on mineral surfaces (2.01∼2.42 kcal/mol) but larger than that of a water molecule adhered on hydrophobic solid surface (0.463 kcal/mol [22]). We assumed that all the atoms within the glutamic acid molecule were at equilibrium with the coarse-grained beads and the distance between the beads and alpha carbons atoms was equal to 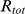. The parameters 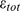 and 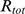 were then utilized to estimate the Lennard-Jones parameters for the coarse-grained beads, 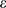 = 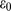 = −0.1177 kcal/mol, and 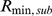 = 0.49 nm at a charge of zero.

### Molecular dynamics simulation

**Figure 2:**
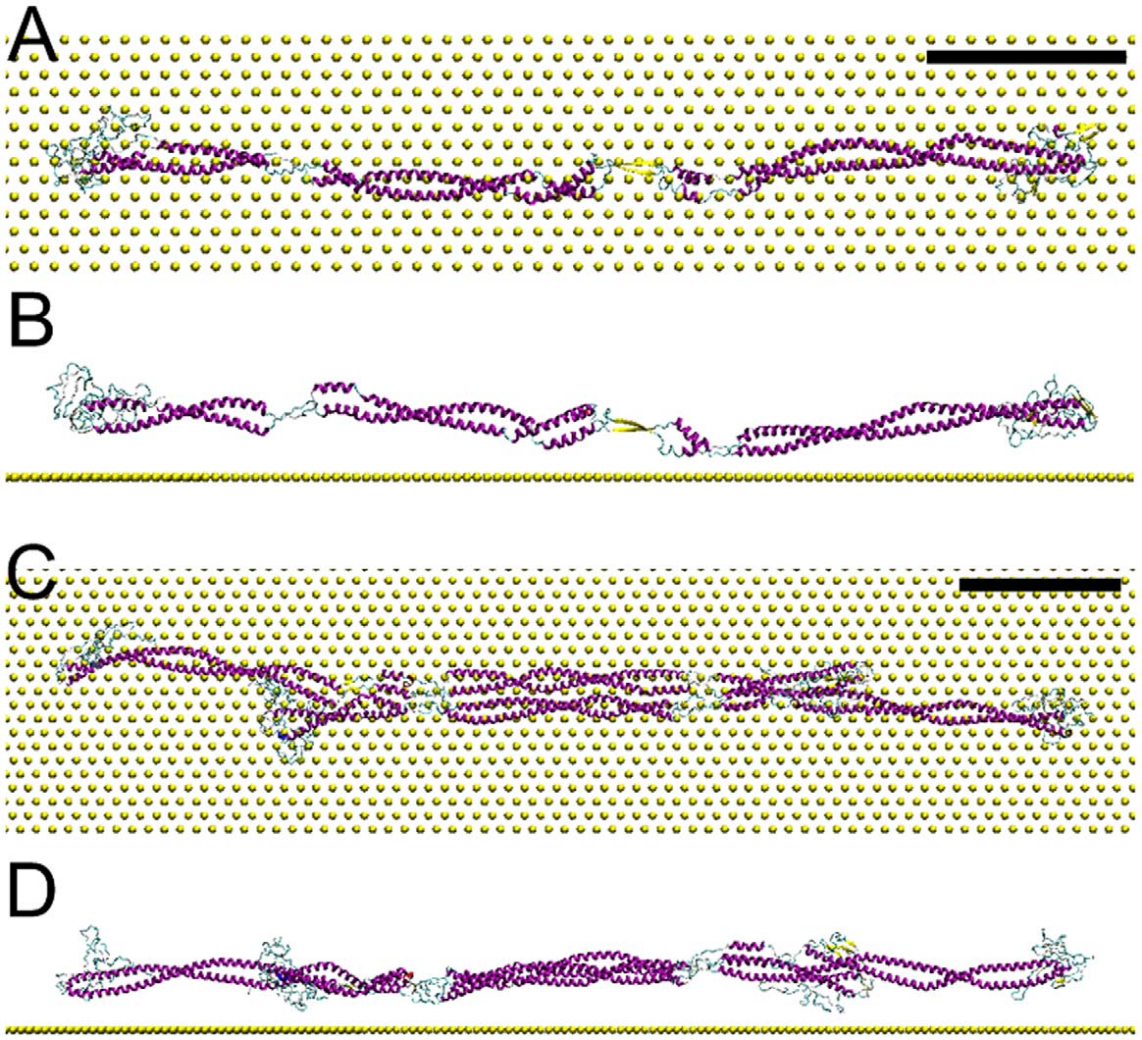
The starting conformation of vimentin subunits in simulation. A) and B) the projecting view and side view of the dimer and substrate, and the scale bar is 10 nm. C) and D) the projecting view and side view of the dimer and substrate, and the scale bar is 10 nm.

The simulated system included a vimentin IF subunit and a coarse-grained silica layer (Figure 2). The initial geometry was taken from [12], using the CHARMM19 all-atom energy force field combined with the effective Gaussian solvent model. We put each of the atomistic models on top of the silica layer with an average distance of 3 nm, which was beyond the cut-off length of the non-bonded interaction. To ensure the deposition of all the subunits onto the substrate, a constant force of 0.0012 kcal/mol/Å^2^ was applied on each amino acid that was more than 5 nm away (vertical distance) from the substrate. The integration time step in simulations is 1 fs and the system temperature is kept at 300 K by the Nose-Hoover algorithm. Silica beads were fixed in space while all the protein atoms were free to move in all directions.

## Results

### Cryo-EM of desmin tetramers and filaments

**Figure 3:**
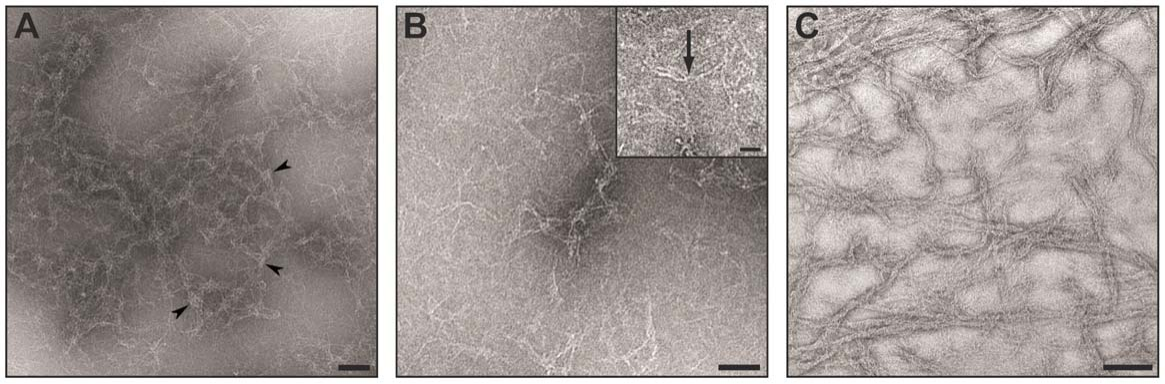
Electron microscopy images of cryo-negative stained samples. A) and B) desmin tetramers. A) the use of a multivalent ion as staining agent induced the formation of networks of tetramers. Notice how the tetramers are bundled together (arrowheads). B) Individual tetramers appear as thin lines (inset, arrow). C) Assembled desmin filaments appear both compact and open. Tetramers are clearly visible in the background. Scale bars 50 nm, inset 20 nm.

**Figure 4:**
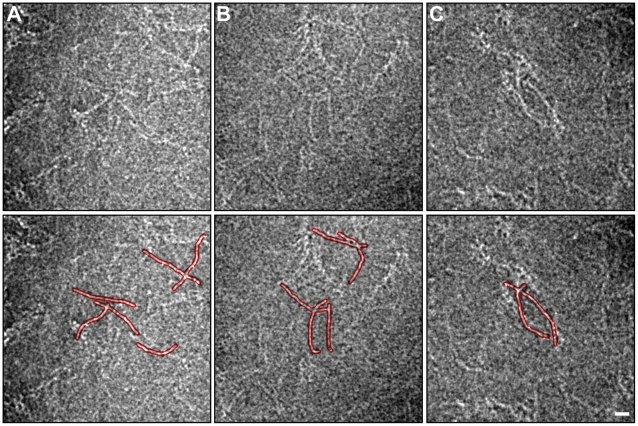
Representative gallery of cryo-negative stained desmin tetramers. In the lower panels the rod shape of prominent tetramers are outlined in red for clarity. Since the images are taken of samples frozen in a layer of vitreous ice approx. 100 nm thick, the depicted tetramers may be overlapping in 3-D. In addition they can be cross linked end-to-end by the staining agent. Scale bar 10 nm.

Subunits of IF were very difficult to image by standard cryo-electron microscopy because of their length and anisotropic shape. This technical difficulty was circumvented by utilizing ammonium molybadate as a cryo-negative stain for the desmin tetramers. As a result, we observed single tetramers embedded in a thin layer of ice (Figure 3A and B). The tetramers appeared as thin smoothly bending rod (Figure 4). However tetramers were also forming apparently two-dimensional networks (Figure 3B). We hypothesize that these networks are induced by the multivalent molybdate ions since they were not observed in unstained preparations. Within the networks, tetramers appeared locally aligned and bundled together (Figure 3A, arrowheads). As expected, these networks were not observed in cryo-stained preparations of assembled desmin filaments (Figure 3C). There were only small difference in the morphology of desmin filaments after cryo-stain or after standard negative staining [23]. Mainly these are differences consists of a better resolution of the filaments sub-structure in cryo-EM vs. standard negative staining where the filaments surface appears smooth with little detail. In addition cryo-EM offers the advantage of observing the tetramers free in 3D without the influence of surface adsorption as well as their detection around compact and open filaments (Figure 3C).

### AFM of dried vimentin and K5/K14 subunits

In order to further characterize the effects of sample preparation on the morphology of IF subunits, we used the AFM to obtain high resolution images of unfixed and dried subunits in various conditions.

**Figure 5:**
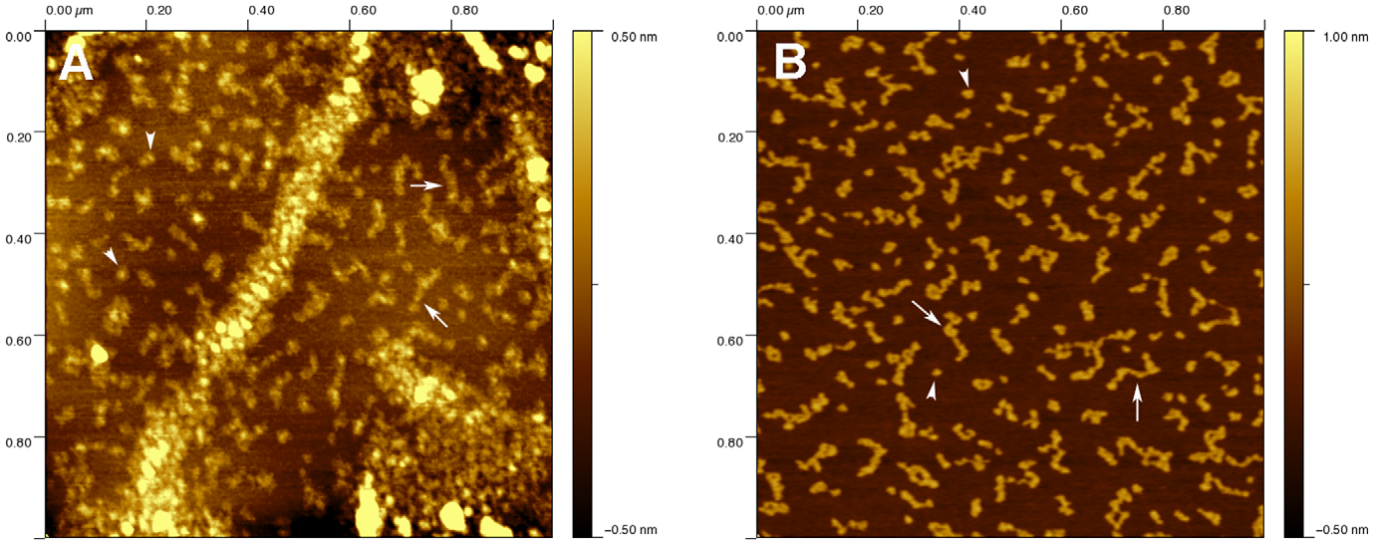
AFM images in air of IFs assembled for 1h at 37°C and absorbed to mica. A) Vimentin at 0.1 mg/ml in 25 mM Tris-HCl (pH 7.5), 125 mM NaCl. Flat filaments are visible as well as vimentin subunits B) Keratin K5/K14 at 0.1 mg/ml in 25 mM Tris-HCl (pH 7.5), 100 mM NaCl. In this picture we only show the IF subunits. In both cases, notice the presence of dot-like structures (arrowheads) along side curly threads (arrows).

**Figure 6:**
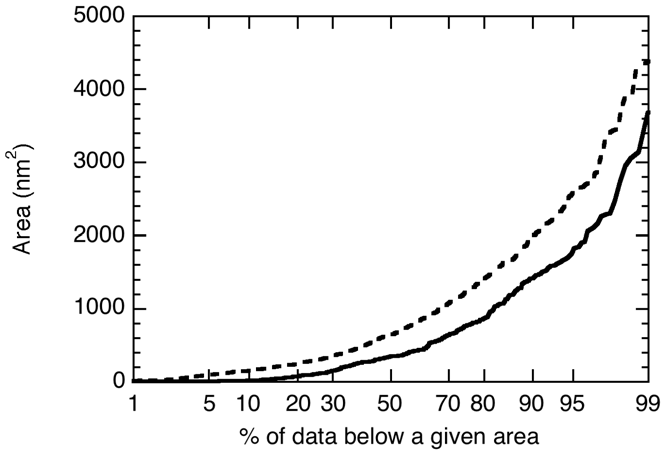
Probability distribution of the area covered by IF subunits presented in **Figure 2**. The solid line corresponds to vimentin and the broken line corresponds to K5/K14.

**Figure 7:**
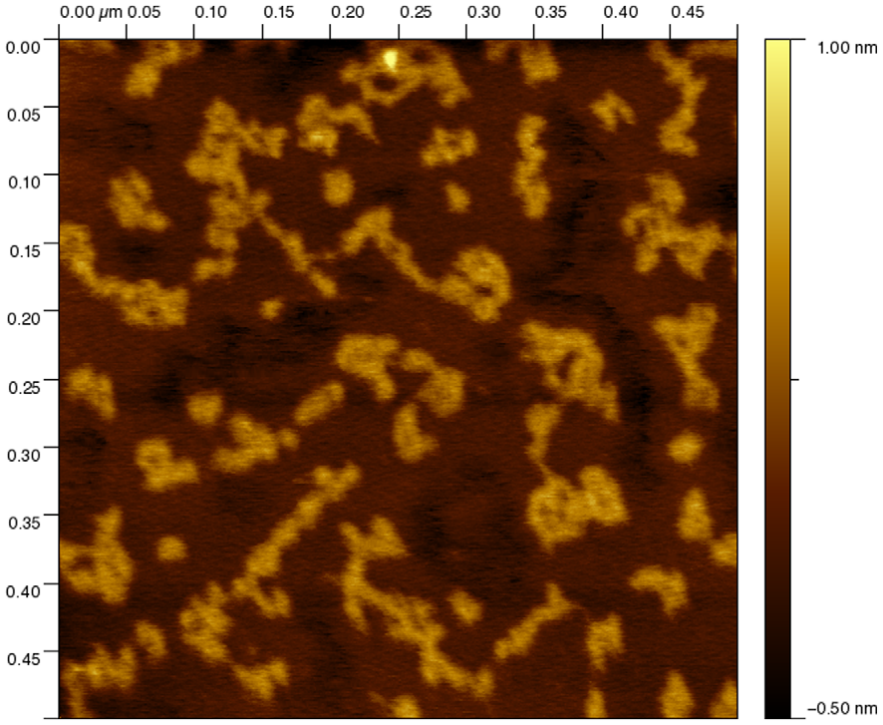
High resolution AFM image in air of K5/K14 subunits after 1h assembly at 37°C in 25 mM Tris-HCl (pH 7.5), 50 mM NaCl. The subunits appear as long coiled threads.

First we imaged vimentin and K5/K14 filaments after one hour assembly at 37°C (Figure 5). In the vimentin case, the filaments appeared wide and flat with an average height of 2.5±0.8 nm (n = 24) and surrounded by IF subunits of different length and conformations (Figure 5A). Such flat tape morphology has already been observed for vimentin filaments absorbed to highly oriented pyrolitic graphite [7]. The vimentin subunits had an average height of 0.31±0.07 nm (n = 58) and covered an average area on the surface of 570 nm^2^. We only analyzed vimentin subunits that were not in contact with filaments. In the K5/K14 case, we either observed compact filaments surrounded by subunits (data not shown) or the subunits alone (Figure 5B). These subunits appeared longer and curlier than the vimentin ones with an average height of 0.62±0.07 nm (n = 67) and an average area on the surface of 909 nm^2^. From our images, it was clear that the subunits take a wide range of conformations on the surface from dots (Figure 5, arrowheads) to curly threads (Figure 5, arrows). However, the distribution of the area occupied by each subunit on the surface did not show any defined peak. Instead we measured distributions that decrease exponentially as if governed by a Poisson process (data not shown). Still, the probability distribution of the area (Figure 6) reveals that keratin subunits (dotted line) tend to occupy a larger area on the surface than vimentin subunits (solid line). We attributed this effect to the presence of longer threads for K5/K14 compared to vimentin as expected from the images (Figure 5). We proposed that these threads may in fact be octameric protofilaments similar to the ones visible in unraveled keratin filaments [24]. This was consistent with high-resolution pictures showing long coiled threads (Figure 7) with an average height double the one of the vimentin subunits (Figure 5A).

**Figure 8:**
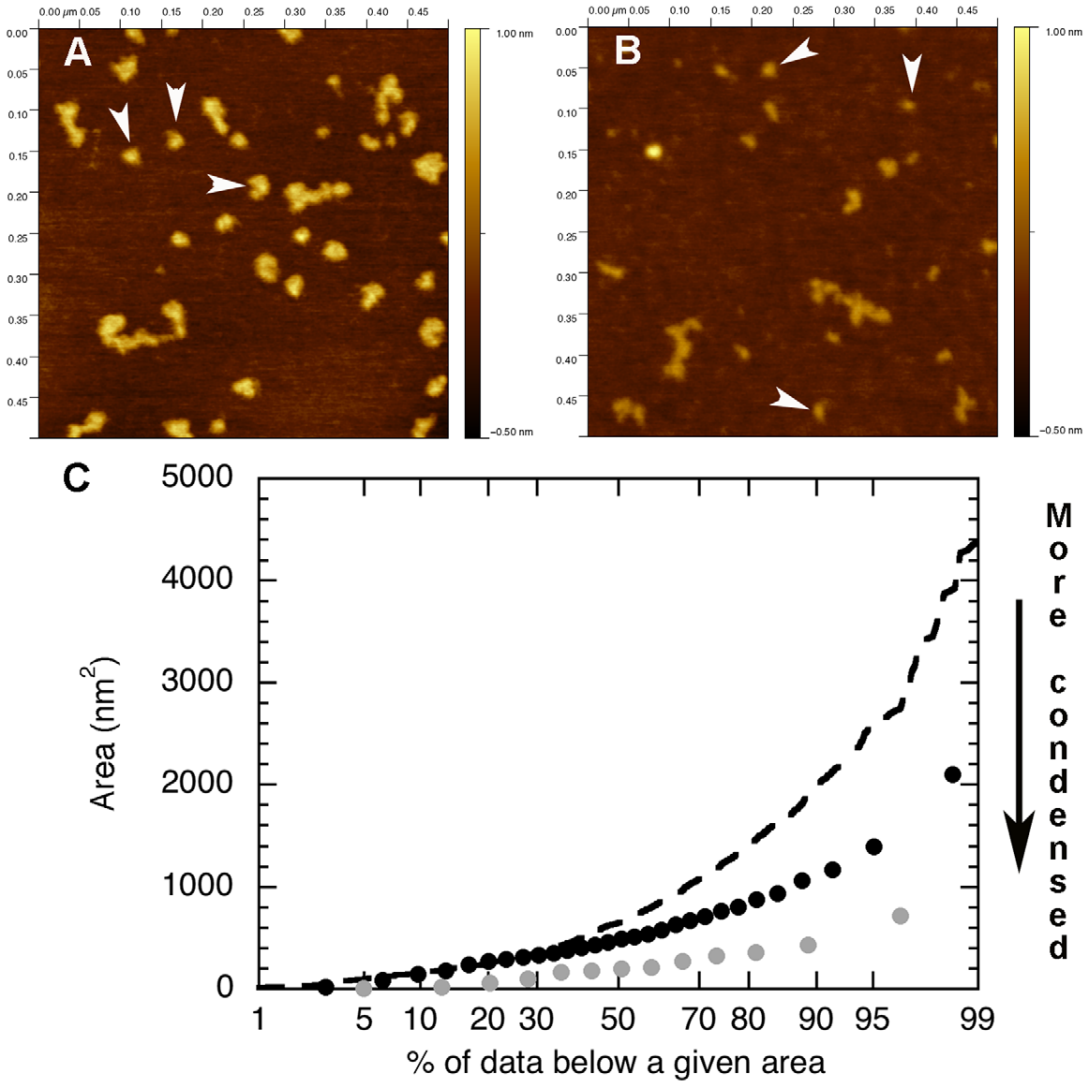
Effect of glutaraldehyde and calcium ions on the conformation of K5/K14 subunits. A) 0.05 mg/ml K5/K14 assembled for 5s at 37°C in 25 mM Tris-HCl (pH 7.5), 50 mM NaCl, quenched with 0.1% glutaraldehyde, absorbed to mica and imaged in air. B) 0.1 mg/ml K5/K14 assembled for 1h at 37°C in 25 mM Tris-HCl (pH 7.5), 5 mM CaCl_2_, absorbed to mica and imaged in air. In both cases, the main subunit population is composed of dots (arrowheads). C) Probability distribution of the area covered by K5/K14 IF subunits. The broken line corresponds to the assembly condition of Figure 2B, the black circles correspond to the glutaraldehyde fixation experiment (A) and the gray circles correspond to the calcium assembly experiment (B).

Second, we analyzed the effect of glutaraldehyde fixation and calcium on these K5/K14 subunits. First, 0.05 mg/ml K5/K14 was assembled for 5 s at 37°C in 25 mM Tris-HCl (pH 7.5), 50 mM NaCl and quenched with 0.1% glutaraldehyde. The subunits appeared mainly as dots with an average height of 0.66±0.07 nm (n = 50) and an average area on the surface of 587 nm^2^ that is equivalent to a disk of diameter 26 nm (Figure 8A, arrowheads). Second, 0.1 mg/ml K5/K14 was assembled for 1h at 37°C in 25 mM Tris-HCl (pH 7.5), 5 mM CaCl_2_. As for the glutaraldehyde case, the subunits appeared as dots with an average height of 0.51±0.06 nm (n = 47) and an average area on the surface of 246 nm^2^ that is equivalent to a disk of diameter 17 nm (Figure 8B, arrowheads). In both cases, the subunits were much more condensed than after NaCl induced assembly. This effect was confirmed when plotting the probability distribution of the area for the three different conditions (Figure 8C).

### Coarse grained MD simulations

**Figure 9:**
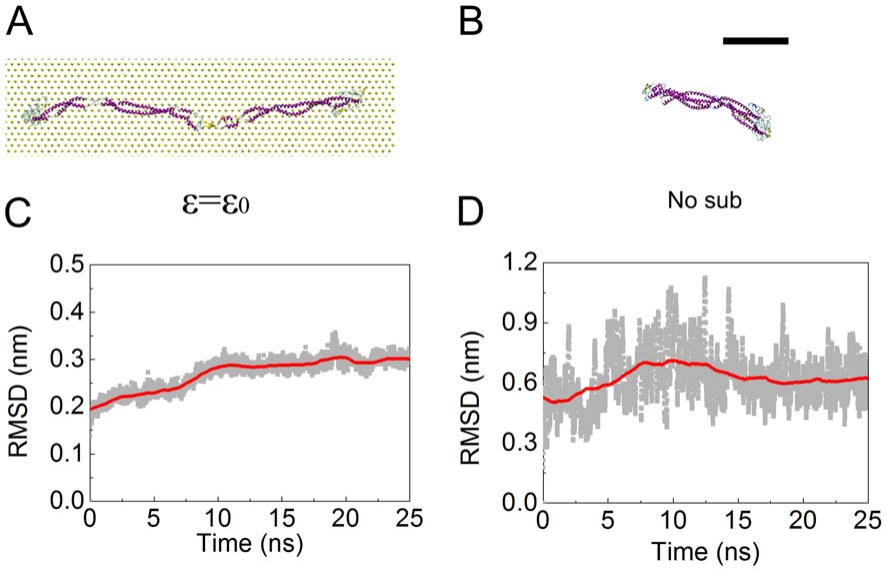
The conformation of vimentin dimers at equilibrium with and without substrate present. A) the equilibrated conformation of the dimer on the substrate with adhesion strength 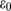. B) the equilibrated dimer without substrate. The scale bar is 10 nm. C) and D) the root mean square deviation (RMSD) of the dimer in (A) and (B) during the last 25 ns in equilibrium simulation, respectively.

**Figure 10:**
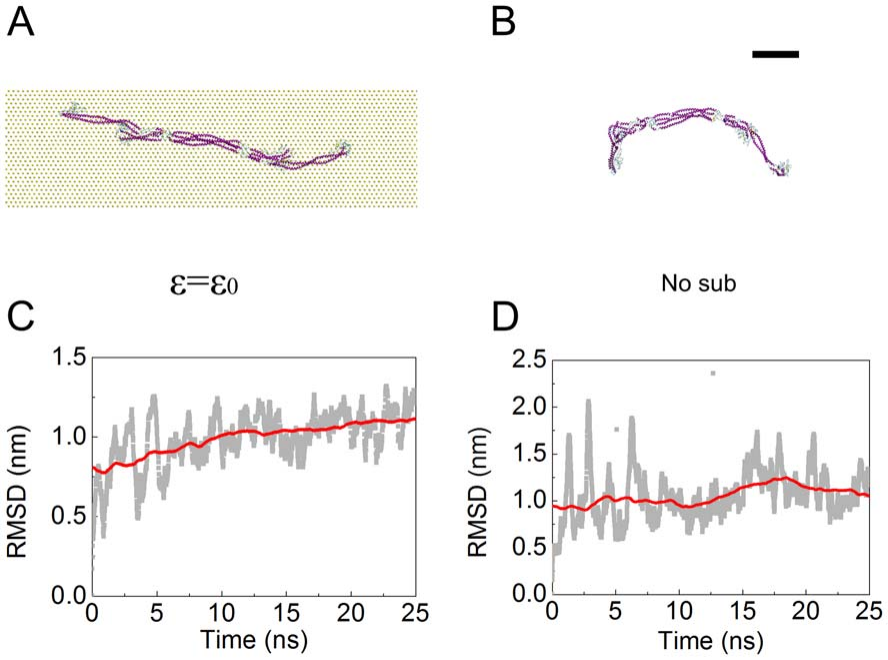
The conformation of vimentin tetramer at equilibrium with and without substrate. A) the equilibrated conformation of the tetramer on the substrate with adhesion strength 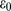. B) the equilibrated tetramer without substrate. The scale bar is 10 nm. C) and D) the root mean square deviation (RMSD) of the tetramer in (A) and (B) during the last 25 ns in equilibrium simulation, respectively.

**Figure 11:**
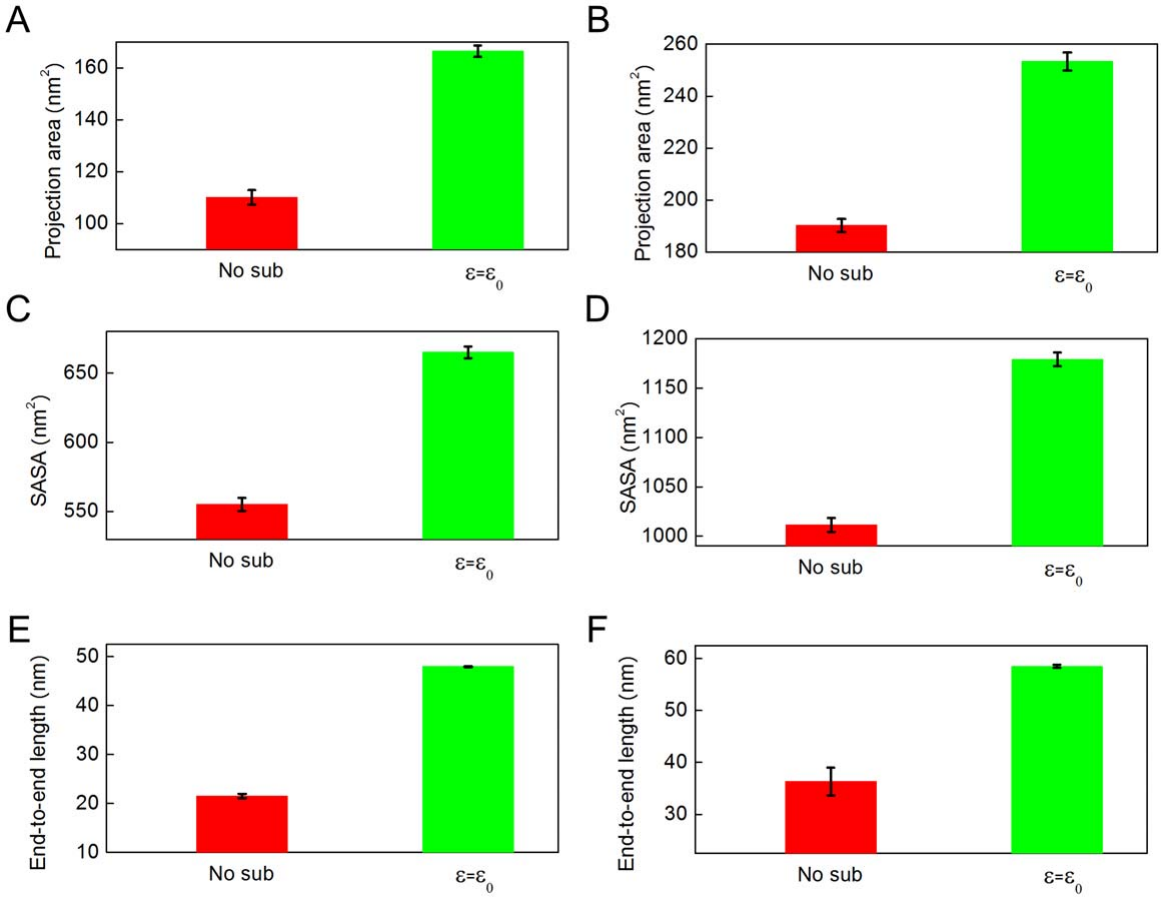
The conformation parameters of the vimentin dimer and tetramer during the simulations with and without adhesion effect comes from substrate. A) and B) the projection area of the dimer and tetramer, respectively. The projecting direction is taken from the first principal axis of the protein model. C) and D) the SASA of the dimer and tetramer, repectively. E) and F) the end-to-end length of the dimer and tetramer, respectively.

In our AFM experiments, we assumed that the conformations of the subunits on mica are very similar to the ones they would have in solution. This hypothesis can be tested *in silico* through MD simulations. We started with the conformations of the vimentin dimer and tetramer presented in Figure 2. After an equilibration time of 50 ns, we observed different conformations of the subunits with and without adhesion energy (Figures 9A, B and, 10A, B). These final conformations were good approximations of the equilibrium state according to the convergence of the root mean square deviation (RMSD) values for each simulation (Figures 9C, D and 10C, D). The main effect of the mica substrate was to keep the subunit conformation in a straighter form compared to its equilibrium state in solution. This could be assessed quantitatively by measuring the projection area, the solvent accessible surface area (SASA) and end-to-end length *L*_ee_ of the dimer and tetramer (Figure 11). *L*_ee_ was the parameter most affected by the presence of the mica. The dimer had an *L*_ee_ of only 21.5±0.5 nm in solution compared to 47.9±0.1 nm on mica (Figure 11E) and the tetramer had an *L*_ee_ of 36.5±2.7 nm in solution compared to 58.5±0.3 nm on mica (Figure 11F). The adhesion energy to the substrate seemed to “freeze” the dynamic properties of the dimer and tetramer. In other words, the surface traps the subunits in their conformation just before adsorption. This means that the difference in morphology between subunits imaged by AFM in air and cryo-negative staining arose from either the capture of short-lived conformations or dehydration effects.

As for the equilibrium conformations in solution, they were in good agreement with experimental data. We already knew from previous studies that the dimer is only soluble in at least 4 M urea [2], so the collapse into a compact 20 nm long rod (Figure 9B) was expected. For the tetramer, the equilibrium conformation showed a smoothly bent shape (Figure 10B) which agreed well with the images of cryo-stained tetramers (Figure 4).

## Discussion

### IF subunits are flexible

All the experimental data presented in this study indicated that depicting IF subunits as straight rods [1], [9], [11] was in fact an over simplification. There is a significant amount of flexibility in IF subunits as seen by cryo-negative staining (Figure 4).

The flexibility of IF subunits is likely to stem from two different structural elements. All IF proteins form either homo- or heterodimers comprising two double stranded coiled-coil segments interspersed by a flexible L_12_ linker [25]. The coiled-coil segments are around 20–25 nm long that is similar to the persistence length of a coiled-coil measured by atomic force microscopy, i.e. 25 nm [26]. Hence, the predicted average angle between the tangents at both ends of one segment is around 70°. The linker L_12_ is predicted to be formed of two poorly structured polypeptide chains [27] and its extreme flexibility has been assessed experimentally for invertebrate IFs [28]. EM of glycerol sprayed and rotary metal shadowed dimers revealed sharp kinks at the L_12_ linkers with angles of 90° and smaller [28]. Our MD simulation of the vimentin dimer (Figure 9) is in perfect agreement with this idea. We observed a folding of the dimer right at the L_12_ linker.

Similar L_12_ kinks have also been observed at the tetramer level [28] which may explain the origin of the 20–25 nm diameter dots that we and others have observed experimentally (Figures 5 and 8) [5], [7]. However, the fact that the ends can touch is not enough to explain the appearance of dots. The coiled-coil segments are negatively charged [2] and will repel each other at high pH. It is clear then that calcium, monovalent salt or a cross-linker is necessary to keep the coiled-coil segment in close proximity. In the physiological case of assembly induced by a monovalent salt such as NaCl, the conserved positively charged arginines in the flexible head domain of IF proteins may mediate dot formation [2], [10] in a transient fashion. These dots were then stabilized by absorption onto mica (Figure 5), by glutaraldehyde fixation (Figure 8A) or by calcium cross linking (Figure 8B).

### Soluble pool and subunit exchange

**Figure 12:**
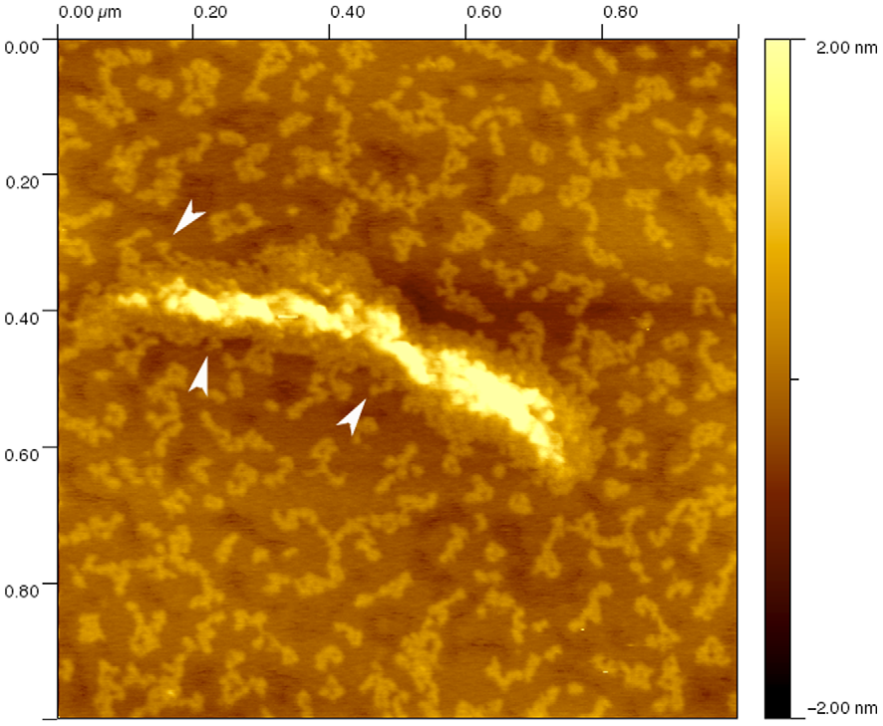
AFM image in air of a K5/K14 filament assembled for 1h at 37°C in 25 mM Tris-HCl (pH 7.5), 50 mM NaCl. This short filament seems to have been caught in the process of spreading onto the surface. Notice how the subunits seem to be radiating outward (arrowheads).

*In vivo*, IFs can exchange subunits with a soluble pool [6], [29]. Several phosphorylation sites [30] control this process. This study demonstrates that a soluble pool of subunits exist along side IFs even one hour after assembly (Figures 3C and 5A). However we do not have any direct proof that subunit exchange can occur *in vitro* in the absence of phosphorylation. Furthermore, the subunits that we imaged by AFM can be dead-end products that are unable to form ULFs and hence remain present throughout assembly. Still, we do have evidence from a previous study that *in vitro* the filaments are dynamic enough to change shape depending on which substrate they are absorbed [7]. By imaging the filaments in air without chemical cross-linking, we have been able to catch compact filaments in the process of spreading flat on a substrate (Figure 12). The subunits can be seen radiating outward from the filament axis (Figure 12, arrowheads) in a way reminiscent of the millipede structure of glycerol sprayed and rotary metal shadowed dephosphorylated neurofilaments [31]. As far as we know, this is the first *in vitro* experimental evidence that subunits can indeed escape from a filament, thus underlying the extreme “plasticity” of IFs and their open polymer nature.

### Conclusion

Using a combination of EM, AFM and MD simulations, we demonstrated that IF subunits are flexible molecules that can adopt a variety of conformations in solution depending on their environment. We hypothesize that switching between extended and compact conformations may be one of the mechanisms used by living cell to regulate the functions of IF proteins, especially their ability to polymerize.

## References

[1] Herrmann H, Bar H, Kreplak L, Strelkov SV, Aebi U (2007). Intermediate filaments: from cell architecture to nanomechanics.. Nat Rev Mol Cell Biol.

[2] Kreplak L, Aebi U, Herrmann H (2004). Molecular mechanisms underlying the assembly of intermediate filaments.. Exp Cell Res.

[3] Herrmann H, Haner M, Brettel M, Ku NO, Aebi U (1999). Characterization of distinct early assembly units of different intermediate filament proteins.. J Mol Biol.

[4] Kirmse R, Portet S, Mucke N, Aebi U, Herrmann H (2007). A quantitative kinetic model for the in vitro assembly of intermediate filaments from tetrameric vimentin.. J Biol Chem.

[5] Herrmann H, Haner M, Brettel M, Muller SA, Goldie KN (1996). Structure and assembly properties of the intermediate filament protein vimentin: the role of its head, rod and tail domains.. J Mol Biol.

[6] Colakoglu G, Brown A (2009). Intermediate filaments exchange subunits along their length and elongate by end-to-end annealing.. J Cell Biol.

[7] Mucke N, Kirmse R, Wedig T, Leterrier JF, Kreplak L (2005). Investigation of the morphology of intermediate filaments adsorbed to different solid supports.. J Struct Biol.

[8] Soellner P, Quinlan RA, Franke WW (1985). Identification of a distinct soluble subunit of an intermediate filament protein: tetrameric vimentin from living cells.. Proc Natl Acad Sci U S A.

[9] Parry DA, Strelkov SV, Burkhard P, Aebi U, Herrmann H (2007). Towards a molecular description of intermediate filament structure and assembly.. Exp Cell Res.

[10] Mucke N, Wedig T, Burer A, Marekov LN, Steinert PM (2004). Molecular and biophysical characterization of assembly-starter units of human vimentin.. J Mol Biol.

[11] Sokolova AV, Kreplak L, Wedig T, Mucke N, Svergun DI (2006). Monitoring intermediate filament assembly by small-angle x-ray scattering reveals the molecular architecture of assembly intermediates.. Proc Natl Acad Sci U S A.

[12] Qin Z, Kreplak L, Buehler MJ (2009). Hierarchical structure controls nanomechanical properties of vimentin intermediate filaments.. PLoS One.

[13] Qin Z, Buehler MJ, Kreplak L (2010). A multi-scale approach to understand the mechanobiology of intermediate filaments.. J Biomech.

[14] Ando S, Nakao K, Gohara R, Takasaki Y, Suehiro K (2004). Morphological analysis of glutaraldehyde-fixed vimentin intermediate filaments and assembly-intermediates by atomic force microscopy.. Biochim Biophys Acta.

[15] Kreplak L, Bar H, Leterrier JF, Herrmann H, Aebi U (2005). Exploring the mechanical behavior of single intermediate filaments.. J Mol Biol.

[16] Herrmann H, Hofmann I, Franke WW (1992). Identification of a nonapeptide motif in the vimentin head domain involved in intermediate filament assembly.. J Mol Biol.

[17] Herrmann H, Wedig T, Porter RM, Lane EB, Aebi U (2002). Characterization of early assembly intermediates of recombinant human keratins.. J Struct Biol.

[18] Adrian M, Dubochet J, Fuller SD, Harris JR (1998). Cryo-negative staining.. Micron.

[19] De Carlo S, Boisset N, Hoenger A (2008). High-resolution single-particle 3D analysis on GroEL prepared by cryo-negative staining.. Micron.

[20] De Carlo S, El-Bez C, Alvarez-Rua C, Borge J, Dubochet J (2002). Cryo-negative staining reduces electron-beam sensitivity of vitrified biological particles.. J Struct Biol.

[21] Autumn K, Sitti M, Liang YA, Peattie AM, Hansen WR (2002). Evidence for van der Waals adhesion in gecko setae.. Proc Natl Acad Sci U S A.

[22] Lee SH, Rossky PJ (1994). A Comparison of the Structure and Dynamics of Liquid Water at Hydrophobic and Hydrophilic Surfaces - a Molecular-Dynamics Simulation Study.. Journal of Chemical Physics.

[23] Bremer A, Henn C, Engel A, Baumeister W, Aebi U (1992). Has negative staining still a place in biomacromolecular electron microscopy?. Ultramicroscopy.

[24] Aebi U, Fowler WE, Rew P, Sun TT (1983). The fibrillar substructure of keratin filaments unraveled.. J Cell Biol.

[25] Strelkov SV, Herrmann H, Aebi U (2003). Molecular architecture of intermediate filaments.. Bioessays.

[26] Schwaiger I, Sattler C, Hostetter DR, Rief M (2002). The myosin coiled-coil is a truly elastic protein structure.. Nat Mater.

[27] North AC, Steinert PM, Parry DA (1994). Coiled-coil stutter and link segments in keratin and other intermediate filament molecules: a computer modeling study.. Proteins.

[28] Geisler N, Schunemann J, Weber K, Haner M, Aebi U (1998). Assembly and architecture of invertebrate cytoplasmic intermediate filaments reconcile features of vertebrate cytoplasmic and nuclear lamin-type intermediate filaments.. J Mol Biol.

[29] Vikstrom KL, Lim SS, Goldman RD, Borisy GG (1992). Steady state dynamics of intermediate filament networks.. J Cell Biol.

[30] Eriksson JE, He T, Trejo-Skalli AV, Harmala-Brasken AS, Hellman J (2004). Specific in vivo phosphorylation sites determine the assembly dynamics of vimentin intermediate filaments.. J Cell Sci.

[31] Hisanaga S, Hirokawa N (1989). The effects of dephosphorylation on the structure of the projections of neurofilament.. J Neurosci.

