# Correction: Plasticity of Intermediate Filament Subunits

**DOI:** 10.1371/annotation/8f182672-e11e-48d5-8a74-c401b8bd5ecc

**Published:** 2010-09-28

**Authors:** Robert Kirmse, Zhao Qin, Carl M. Weinert, Andreas Hoenger, Markus J. Buehler, Laurent Kreplak

The fourth author's name is spelled incorrectly. The correct name is: Andreas Hoenger. 

